# Draft Genome Sequence for Klebsiella michiganensis B199A, Originally Identified as Enterobacter aerogenes

**DOI:** 10.1128/mra.00310-22

**Published:** 2022-05-18

**Authors:** Matthew J. Igo, Donald W. Schaffner

**Affiliations:** a Department of Food Science, Rutgers University, New Brunswick, New Jersey, USA; University of Maryland School of Medicine

## Abstract

Here, we report the draft genome sequence of a strain of Klebsiella michiganensis originally identified as Enterobacter aerogenes B199A. This strain has been used as a Salmonella surrogate to study the effectiveness of handwashing and measure cross-contamination to and from a wide variety of surfaces and foods.

## ANNOUNCEMENT

We report the draft genome sequence of Klebsiella michiganensis strain B199A originally identified as Enterobacter aerogenes. B199A was first used to study microbial cross-contamination in the kitchen (1) and originally obtained from Vivolac Cultures, Greenfield, Indiana. Commercial use was for fermenting egg white prior to drying to reduce Maillard browning (2), and the original source was likely naturally fermented egg whites (3). Ribotyping of B199A by Vivolac in 2017 identified it as Klebsiella pneumonia, and they discontinued its sale (personal communication was from WD Sing). We have used this culture to study handwashing (4), cross-contamination to and from hands (5) and surfaces (6), and survival on different surfaces (7).

B199A was revived from our culture collection on tryptic soy agar with nalidixic acid (TSA-na) (Fisher Scientific, Waltham, MA) from −80°C and incubated at 37°C for 24 h. A single colony was transferred into 10 mL of TSB-na (Fisher Scientific) for 24 h at 37°C. Genomic DNA was extracted using the Promega (Madison, WI) Wizard DNA extraction kit (https://www.promega.com/resources/protocols/technical-manuals/0/wizard-genomic-dna-purification-kit-protocol/). Extracted DNA was refrigerated and sent for sequencing (Genewiz, South Plainfield, NJ). Results were returned as raw FASTQ files. Illumina MiSeq 2 × 150-bp sequencing was used for the sequencing with coverage depth set to 50× using pre-existing Enterobacter data. An analysis was conducted on GalaxyTrakr with default parameters unless otherwise stated. Trimmomatic v. 0.38 (8) using the SLIDINGWINDOW default parameters removed adapter sequences. Sequence reads were *de novo* assembled using SPAdes v. 3.12.0 (9) with k-mers autodetected as 31, 45, 59, 73, and 87. The average read length of the sequence was 120 bp. QUAST v. 5.0.2 (10) determined statistics on the assembled genome. AMRFinder v. 3.8.28 (11) determined antimicrobial resistant genes. FastANI v. 1.33 (12) analysis was performed against *Enterobacteriaceae* until it was determined that the strain was *K. michiganensis* based on an average nucleotide identity (ANI) value of 98.7. This result was confirmed upon deposit to GenBank. *K. michiganensis* B199A had a sequence length of 6,117,020 bp, with trimmed reads containing an average of 120 bp and a total read count of 7,034,327 for a coverage of 138× with a GC content of 55.65%. The genome contained 72 contigs with an *N*_50_ of 212,765 bp. Antibiotic-resistant genes include a class A extended-spectrum beta-lactamase, OXY-1-1, and an aminoglycoside *O*-phosphotransferase, *aph(3′)-Ia*. Assembled sequences were annotated using the NCBI Prokaryotic Genomes Annotation Pipeline (PGAP) v. 6.0.

### Data availability.

The sequences were deposited in GenBank under SRA accession number SRR18059839 and BioProject PRJNA807804. This whole-genome shotgun project was deposited at DDBJ/ENA/GenBank under the accession JAKSGB000000000.
